# Does the relationship marketing orientation of an entrepreneur support agency improve performance? Evidence from small- and medium-size enterprises in Malaysia

**DOI:** 10.1371/journal.pone.0269319

**Published:** 2022-06-16

**Authors:** Nor Asiah Omar, Hasnan Md. Aris, Muhamad Azrin Nazri, Taslima Jannat, Syed Shah Alam

**Affiliations:** 1 Faculty of Economics and Management, Center of Value Creation and Human Well-being Studies, Universiti Kebangsaan Malaysia, Selangor, Malaysia; 2 Higher Education Division Majlis Amanah Rakyat (MARA), Kuala Lumpur, Malaysia; 3 Faculty Economics and Muamalat, Universiti Sains Islam Malaysia, Nilai, Malaysia; 4 UKM-Graduate School of Business, Universiti Kebangsaan Malaysia, Selangor, Malaysia; Universiti Pertahanan Nasional Malaysia, MALAYSIA

## Abstract

Entrepreneur support agencies are highly important in the development of small-and medium-size businesses of entrepreneurs. There are a number of studies on support agencies, but studies on entrepreneurial performance from the perspective of a relationship marketing orientation (RMO) between support agencies and entrepreneurs are lacking. This study aimed to investigate the hypothesized relationships between the RMO of an entrepreneur support agency and the financial and nonfinancial performances of small- and medium-size entrepreneurs (SMEs). A total of 276 valid SMEs survey responses based on purposive sampling were collected and analyzed using partial least squares structural equation modeling (PLS-SEM). Findings indicate that trust and reciprocity are the significant factors to financial performance of SMEs. Meanwhile, trust, communication, empathy, and reciprocity indicate a significant positive relationship with nonfinancial performance of SMEs. To the best of the authors’ knowledge, this is the first study to provide an interesting avenue to understand the relationship between an entrepreneur support agency and entrepreneurs to work on synergistic relationship approaches in order to remain sustainable in the market. This study has also drawn specific implications for SMEs and government agencies for entrepreneur and policy planning to coordinate appropriate entrepreneurship development programs and strengthen the entrepreneurship ecosystem.

## Introduction

Small- and medium-size entrepreneurs (SMEs) are essential in boosting the economies of most countries. For this reason, governments, agents that support entrepreneurship activities, and other organizations around the world have offered support programs for enhancing the performance of SMEs. Such programs offer entrepreneurs training and information on a number of aspects of business development, including increasing the scope of financing for business expansion and innovation, developing information technology tools, accessing networks, and reducing the regulatory burden. The program aims to encourage entrepreneurs’ performance, sustainability, innovation, productivity, and employment generation [1,2] through triple-helix programs [3,4] that coordinate the efforts of governments, universities, and industries. The support agencies are essential in helping SMEs to develop, particularly in terms of improving their business performance [5–7]. Prior studies have examined several aspects of entrepreneur support programs, such as grants for individuals [8] and firms [9–11], as well as training grants [12–14]. Several other studies have covered mentoring [15], financial assistance [16–19], institutional support and resource [20], and training [1,2].

Although much support has been given to SME development and enhancement, it is still unfortunately the case that many SMEs fail or perform poorly in Malaysia [21,22] and other parts of the world. Most of the existing studies on entrepreneur support are merely phenomenological studies or use secondary data related to the motivations of entrepreneur support agencies [23] and impact of government business support on SMEs performance [7]. The review of extant research related to entrepreneur support and SMEs reveal inconsistency findings that warrant further study. One such gap is the critical gap in the literature on the connections between each entrepreneur support initiative and its outcomes. For instance, recent findings suggested that government support program is related to firm performance [7] in contrast Westhead and Birley (1995) argued that entrepreneur support program has no impact on SMEs performance despite it is often used to relieve SMEs weakness’ [24]. Although much planning and effort and many activities take place between entrepreneur support agencies and entrepreneurs to promote the entrepreneur’s success, few studies have investigated the role of support agencies in an entrepreneur’s performance from the perspective of RMO. As entrepreneurial success is never wholly predictable and the allocation of resources will always vary to firms, several scholars have pointed the critical need to study the heterogeneous treatment effects of entrepreneur’s support program to firm outcomes [25,26].

Past studies discovered that RMO influences firms in terms of their long-term performance [27,28], customer retention (CR) [29], marketing effectiveness [30], and brand equity [31], but no research has analyzed RMO from the entrepreneur support agency perspective. Ongoing interactions and interpersonal communications between customers and service providers are considered very important because they serve as determinants of the success of service delivery [32,33]. Accordingly, in the context of family firms stewardship attitudes and behaviors among owners and leaders deliver better outcomes and performance [34].

Venkatraman and Ramanujam [35] emphasized the need to acknowledge two different perspectives on business performance, namely,

- financial performance, which reflects a strategic management perspective and organizational effectiveness covering the company’s economic goals, such as sales growth and profitability, and
- nonfinancial performance, which emphasizes operational performance indicators other than financial performance indicators, such as product quality, marketing effectiveness, and customer retention (CR).

Similarly, Wiklund and Shepherd [36] suggested that a broad business performance includes not only financial performance but also nonfinancial performance. The fit of both financial performance and nonfinancial performance with the components of the entrepreneur support agency RMO resulted in these two variables being included in the examination of the components of RMO in the current study. Despite a number of research studies examining the outcomes of RMO, there are notable gaps in the literature on the empirical testing of the relationship between a support agency and an entrepreneur and how this relationship affects a firm’s performance in terms of the two perspectives of financial performance and nonfinancial performance.

In addressing the research problem, this study explored the application of the resource-based view theory [37] and the relationship marketing theory [38] in the field of entrepreneurship. Resources can be tangible (e.g. capital, building, inventory) or intangible (e.g. reputation, knowledge, relational capital) in nature [39]. A study on SMEs and business-to-business commerce suggested the importance of building a relationship with and collaborating with the client to generate and improve financial performance [40]. According to the resource-based view theory, government support policies are essential for SMEs in the early stages of their life cycles to help SMEs achieve better company performance [41]. Furthermore, to curb the issue of limited resources, SMEs can optimize their value creation by working with strategic partners to leverage their competitive advantage and improve their performance in the long run [42].

Based on the issue highlighted, this study aimed to test the hypothesized relationships between the components of entrepreneur support agency RMO with SMEs’ financial performance and nonfinancial performance. To our knowledge, this is the first study to relate RMO entrepreneur support agency to financial and nonfinancial performance of SMEs. The rest of this paper is structured as follows. The next sections of this paper are the literature review and hypotheses development, the methodology used, the results of the study, along with a discussion of them and their implications, the limitations of the study, and suggestions for future research.

## Literature review and hypotheses development

### Firm performance

Doyle [43] argues that a company’s performance cannot be guided by a single measure because each firm uses different objectives and measurements to evaluate its performance. Firm performance should be measured as a win‒win situation. A firm should be able to achieve its goals and objectives while fulfilling its stakeholders’ needs. Prior studies illustrate various methods that have been used to measure performance [44]. Venkatraman and Ramanujam [35] highlighted that performance can be measured through financial performance, business performance, and organizational effectiveness. Specifically, organizational effectiveness refers to organizational performance that includes the introduction of new products, product quality levels, value-added production processes, and marketing effectiveness. Venkatraman and Ramanujam [35] proposed to use operational measurement to compliment financial measurement in representing firm performance. According to this argument, a company’s performance should be viewed from two different perspectives, namely, the achievement of financial performance and nonfinancial performance [45,46]. Financial performance entails the market performance in terms of the sales volume and high market ratios, as well as the profit margin and return on investment [47,48]. In a much-related context to this study, the sales, sales growth, net profit, and growth profit are among the financial measures favored by the SMEs in Malaysia [49]. The non-financial perspectives are marketing issues such as customer satisfaction scores for measures of product or service quality [50]. They are alternative indicators of organizational effectiveness. Usually, they include a subjective qualitative performance, such as the quality of customer service, marketing effectiveness, strategy achievement, employee satisfaction, corporate culture and customer retention [51,52].

### Financial performance and nonfinancial performance

Firm performance is often measured by the financial success of an organization. For profit-oriented firms, financial success can be assessed through the “top line” (e.g., sales) and “bottom line” (e.g., profitability) [53]. In measuring financial profitability, the most common measurements are profit margin, return on assets, return on equity, return on investment, and return on sales [54,55]. According to Hernaus, Bach, and Vukšić [56], the nonfinancial performance should be included in measuring the performance. The measurement of nonfinancial performance can be assessed through customer satisfaction, customer loyalty, employee turnover, and customer retention [57,58]. Thus, for this study, non-financial performance is measured as customer retention (CR) [51].

CR is the activity of engaging existing customers to continue a business relationship through excellent customer service that enhances long-term customer satisfaction [59,60]. Jeng and Bailey [61] highlighted the importance of a long-term business relationship, which can be realized through non formal or formal activities. CR is considered an essential determinant in measuring firm performance for an SME primarily to ensure the sustainability of the firm [62].

### Relationship marketing and relationship marketing orientation

The popularity of relationship marketing begins with the convergence of various factors, such as the transition to service-based economics, communication, technology advancement, logistics, and global competition [63]. The definition of relationship marketing was gradually improved from year to year. Berry [38] defined relationship marketing as “attracting, maintaining, and enhancing customer relationships” (p. 25). The meaning has been further expanded, incorporating the need to exercise a mutual exchange and fulfill promises [64].

Sheth and Parvatiyar [65] describe relationship marketing as an inclusive activity involving customers, suppliers, and other industry partners to expand a firm’s development and marketing activities. Gummesson [66] defined relationship marketing as relationships, networks, and interactions. In comparison, [67] suggested that relationship marketing involved the activities of identifying, establishing, maintaining, and enhancing relationships with customers and other stakeholders to maximize profits and meet the objectives of all parties through a mutual exchange and the fulfillment of promises. New business practices and thinking have evolved from marketing orientation to relationship marketing orientation (RMO), according to Callaghan [88]. RMO focuses on maintaining and building mutual reciprocal, trust and ties between two parties in an exchange, i.e. the seller and the buyer [88].

The most recent researchers have proposed that relationship marketing be measured according to a few factors. East, Hammond, and Gendall [68] further explained relationship marketing as attention to retaining customers by producing quality improvement. Additionally, Hunt, Arnett, and Madhavaram [69] identified six factors that are associated with the successful relational exchange, namely, trust, commitment, keeping promises, cooperation, communication, and shared values. The assumption is that these six factors will lead to relationship marketing success in terms of competitive advantage, financial performance, satisfaction, learning, propensity to stay, acquiescence, and a decrease in uncertainty.

### Relationship marketing orientation (RMO) and performance

Based on the definitions in the relationship marketing literature, RMO is measured using six dimensions, namely, trust, bonding, communication, shared values, empathy, and reciprocity [70]. This scale has been used in previous studies (e.g., [31,71]) to examine the relationship between RMO and company performance.

In line with previous literature, this study adopted the definition of RMO by [27] indicating that RMO embraces the creation and maintenance of relationships between two parties (support agencies and entrepreneurs) in an exchange, with an emphasis on the development of empathy, reciprocity, trust, and bonding.

RMO has been studied quite extensively for industries such as manufacturing, finance, hotels, and retailing [72–75]. Several past studies discovered that RMO influenced firms in terms of business performance [28], marketing effectiveness [30], brand equity [31], positive word of mouth [76], identification [77], and customer loyalty [78]. Accordingly, other studies (e.g., [79–83] have demonstrated the positive relationship between RMO and firm performance.

### Trust

Trust remains an essential element of business relationships, especially in the consumer and business markets [84]. Trust plays a vital role in enhancing long-term relationships and loyalties [85] and CR [86,87]. It is construed as the component of a business relationship that establishes the manner to which each party feels it can depend on the credibility of the other party [88]. Trust is a reciprocity behavior that is theorized to maximize the probability of established long-term relations between parties [27]. A close inter firm relationship based on trust reduces the perceived vulnerability between parties [89]. In retailer and supplier relationships, the establishment of trust between the parties positively influences the strategic performance and financial performance. Trust has an indirect significant effect on customer retention (CR) through satisfaction. A study by [90] found that trust is the key driver of CR. In line with the above discussion, we propose the following hypothesis for testing:

*H1a*: *There is a positive relationship between the entrepreneur support agency relationship marketing orientation (RMO) (trust) and financial performance (FP)*.

*H2a*: *There is a positive relationship between the entrepreneur support agency relationship marketing orientation (RMO) (trust) and nonfinancial performance (CR)*.

### Bonding

Bonding is defined as the dimension of a business relationship that results in two parties (buyer and seller) acting in a unified manner toward a desired goal [88]. A long-term buyer‒seller relationship results in strong personal bonding between the parties and develops a better commitment to retain the relationship [27,91]. Several researchers have also suggested that bonding is positively related to components of firm performance such as market share and CR [27,75].

Through bonding ties, participating partners can generate attainable ideas and resources that contribute to better product development and innovation, in turn enhancing a firm’s sales growth [92–94]. The activities that take place through the bonding relationship help a firm to make sensible decisions and, thus, achieve a better firm performance [95]. In addition, financial, social, and structural bonding efforts positively affect CR through customer loyalty [96]. Consequently, it can be hypothesized that

*H1b*: *There is a positive relationship between the entrepreneur support agency relationship marketing orientation (RMO) (bonding) and financial performance (FP)*.

*H2b*: *There is a positive relationship between the entrepreneur support agency relationship marketing orientation (RMO) (bonding) and nonfinancial performance (CR)*.

### Communication

Communication can be defined as both the formal and informal exchanges of useful and beneficial information between buyers and sellers [27,97]. A successful business relationship has crucial components of cooperation and trust, which develop from excellent communication behaviors [98]. In another point of view, empirical evidence suggests that communication improves the level of trust between partners [97–99] and increases CR [29,100].

In the business environment, communication is the core practice in maintaining the interest of various stakeholders [101]. Communication encourages information sharing, and this has a positive impact on the benefits of a relationship, the authorizations of customers, and the commitments of a relationship [102]. Two-way communication is an effective way for both parties to share ideas and benefits for a positive firm performance [103]. In line with the above discussion, we propose the following hypothesis for testing:

*H1c*: *There is a positive relationship between the entrepreneur support agency relationship marketing orientation (RMO) (communication) and financial performance (FP)*.

*H2c*: *There is a positive relationship between the entrepreneur support agency relationship marketing orientation (RMO) (communication) and nonfinancial performance (CR)*.

### Shared values

Shared values is the degree to which partners own shared beliefs about what actions, objectives, and policies are essential, acceptable, and correct [98]. It is believed that the shared values of parties can increase the level of commitment in business relationships [98]. When participating partners have common values or beliefs, they will be more invested in their relationship, and this will positively affect their performance [27]. Kwan and Carlson (2017) [75] defined shared values as a similarity of beliefs between two parties in a transactional exchange. They opined that a firm manages to achieve greater financial and nonfinancial performances when the parties can coordinate their preferences to encourage harmonious conflict solution. Based on the above information, it can be hypothesized that

*H1d*: *There is a positive relationship between the entrepreneur support agency relationship marketing orientation (RMO) (shared values) and financial performance (FP)*.

*H2d*: *There is a positive relationship between the entrepreneur support agency relationship marketing orientation (RMO) (shared values) and nonfinancial performance (CR)*.

### Empathy

Empathy is seen as a critical condition for fostering a healthy relationship between two parties. [27] discussed its inclusion in the literature on service marketing and networks. Empathy is described as attempting to ascertain someone else’s desires and aims, in this case, those of a client [70]. In a relationship situation, empathy is the ability of a person to express, understand, and feel the feelings of the other [104].

In a much-related context, when financial providers such as banks and SMEs have a close interpersonal relationship and understand each others’ values and goals, this will contribute to a better firm performance. The decisions made will benefit both parties [73] An organization that practices empathy through RMO has a different relationship to its customers than other organizations from the perspectives of its customers, and this causes the organization to retain its clients. Therefore, it is hypothesized that

*H1e*: *There is a positive relationship between the entrepreneur support agency relationship marketing orientation (RMO) (empathy) and financial performance (FP)*.

*H2e*: *There is a positive relationship between the entrepreneur support agency relationship marketing orientation (RMO) (empathy) and nonfinancial performance (CR)*.

### Reciprocity

According to [27], reciprocity is an element of the business relationship that allows either party to grant favors or make allowances. This concept is well known and is frequently referred to as investments unique to relationships [105]. Jayachandran et al. [106] defined reciprocity as a mechanism allowing customers to communicate with, exchange information with, and enable the company to respond to customers. It is concluded that consumer reciprocity is important for future business revenues [107]. Past studies also found that reciprocity is related to behavioral loyalty [108] and customer satisfaction [109]. The ability of a firm to reciprocate with customers eventually increases switching costs and develops CR [110]. Based on RMO, reciprocity influences a firm’s market share, sales growth, CR, and return on investment [75] Consequently, it can be hypothesized that

*H1f*: *There is a positive relationship between the entrepreneur support agency relationship marketing orientation (RMO) (reciprocity) and financial performance (FP)*.

*H2f*: *There is a positive relationship between the entrepreneur support agency relationship marketing orientation (RMO) (reciprocity) and nonfinancial performance (CR)*.

**Fig 1:**
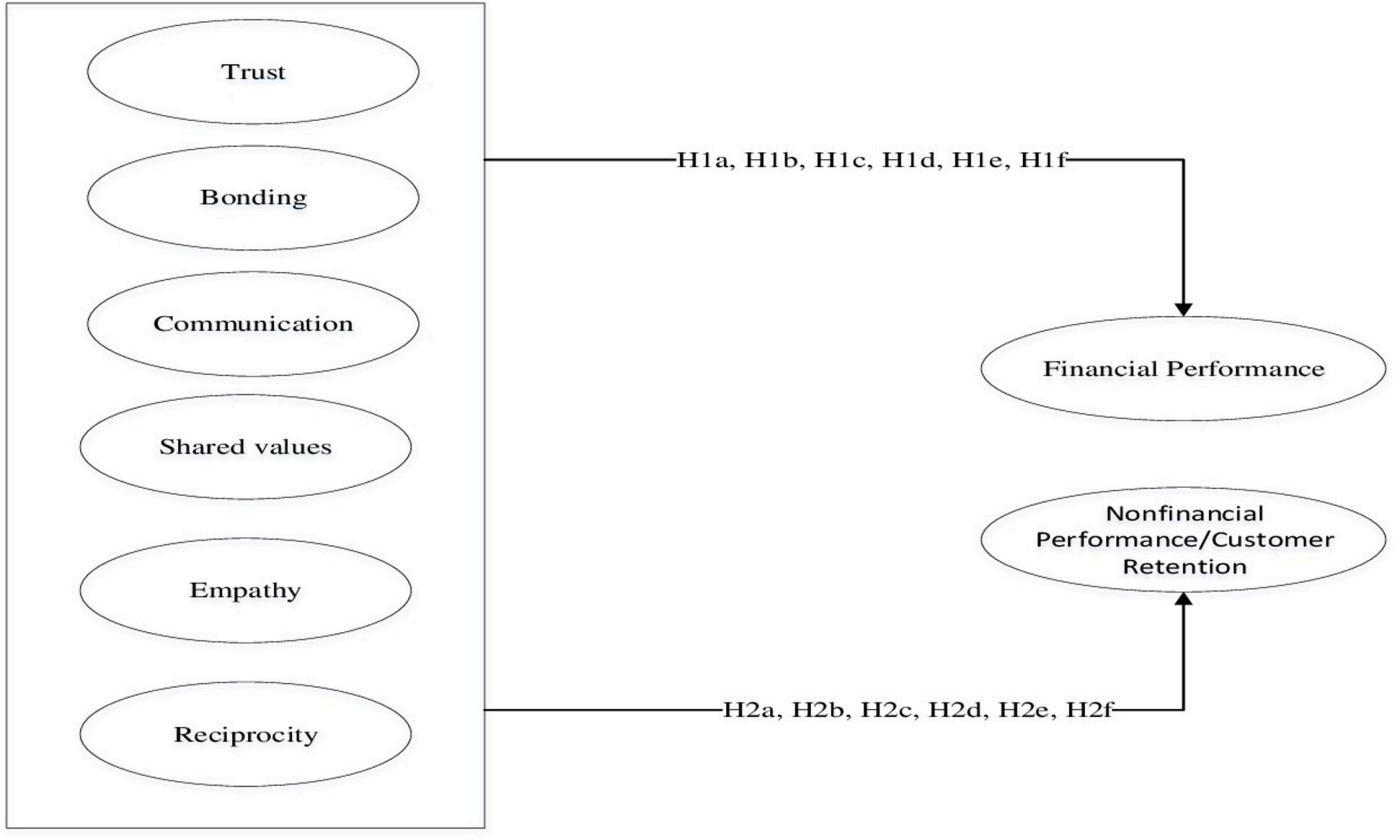
Conceptual framework.

Fig 1 shows the study’s conceptual framework and the hypothesized relationships.

## Methodology

### Research design and data collection procedures

A survey was conducted to examine the hypotheses in this study. The research scope was narrowed to the organizational level. The questionnaires were distributed to 500 SMEs in Kuala Lumpur and Selangor through purposive sampling. A personally administered questionnaire method was used for data collection. In ensuring the survey’s reliability, a back-translated method was used to translate the English version into Bahasa Melayu (the Malaysian national language). The questionnaire was first pretested among 10 SMEs and 3 academicians in Kuala Lumpur. Based on the feedback of the respondents, a few changes were made to the questionnaire’s wording before the questionnaire was finalized.

The respondents were approached in a training and seminar room of the entrepreneur support agency’s building. The sampling frame was entrepreneurs (i.e., owners, senior managers, chief executive officers of SMEs) who had operated a business for at least 1 year and had received assistance from an entrepreneur support agency in Kuala Lumpur or Selangor. The type of assistance received varied according to the type of program in which the SME had participated. People with higher positions in an SME (i.e., those involved in decision making and company strategy) are most commonly involved in entrepreneur support programs. The objectives of the research were first explained to the entrepreneurs. They were guaranteed strict confidentiality for the answers on their returned questionnaires. Statistical analyses were performed with SmartPLS 3.2.8 software using structural equation modeling techniques and the partial least squares (PLS) method [111].

The researchers carefully reviewed the responses for each question. Inappropriate responses, such as answers that were similar for all questions and incomplete answers, were omitted from the sample. Out of a total of 297 questionnaires collected, only 276 were used for further analysis. This indicated a 75% response rate, and that can be considered an adequate sample in the field of research involving SMEs. This level of usable responses was comparable to that of similar survey-based studies involving SMEs in Malaysia [112–114]. These valid responses were assessed for reliability, validity, and appropriateness for hypotheses testing.

### Measures

The questionnaire consisted of two parts, the constructs and the demographic profile (company size, expertise and market form, age, and gender), to ensure complete anonymity. Both constructs were calculated using multi-item elements and modified with minor changes from previous studies (see Appendix A). The measurement scales used in the questionnaire included aspects that reflect RMO (i.e., trust, bonding, shared values, communication, empathy, and reciprocity). RMO refers to attracting, preserving, and developing relationships through the mutual sharing and fulfilling of agreements between entrepreneur support agencies and entrepreneurs [38,64]. All the constructs were measured by a seven-point scale, from 1 = strongly disagree to 7 = strongly agree. The financial and nonfinancial performances were measured by four items and seven items, respectively. The seven survey items for CR (nonfinancial performance) were drawn from the extant literature [e.g., 115–117]. The respondent-entrepreneurs were asked to indicate the extent to which their customers are putting an effort to maintain a business relationship with them by repeating their usage behavior using a seven-point scale (1 = strongly disagree to 7 = strongly agree). Given that the sample focuses only on SMEs, this study uses subjective performance measures on performance [see 34]. The respondents’ perceptions of the four financial performance items drawn from previous research [118,119], that is, return on investment, cost, profit, and sales, were collected. All four items for financial performance were rated on a seven-point scale ranging from 1 = much worse to 7 = much better. The respondents were asked to compare the company’s financial performance in previous years with the performance of similar SMEs in the industry.

## Results

### Demographic profile of the respondents

Table 1 shows that the sample was slightly dominated by women entrepreneurs (women, 52.1%, and man, 47.9%). The data show that more women were involved in these businesses, and these statistics can be generalized to all other cities in Malaysia. In terms of age distribution, 34.5% of respondents were age 30 to 39 years, 29.3% were 20 to 29 years, 20.4% were 40 to 49 years, and 10% were 50 to 59 years. The entrepreneurs had different types of businesses, with 51.5% having sole proprietorships, 35.9% having partnerships, and 12.6% having private limited companies. There were 161 respondents (58.5%) who owned their businesses. Of the respondents, 103 (37.4%) earned an income of RM10,000 to RM100,000 and 93 (38.9%) earned an income of RM100,001 to RM200,000.

**Table 1:** Survey respondent profile (n = 276).

Measure	Item	Frequency	Percentage (%)
Gender	Male	132	47.9
	Female	144	52.1
Age	Under 20	9	3.1
	20–29 years	81	29.3
	30–39 years	95	34.5
	40–49 years	56	20.4
	50–59 years	28	10.0
	60 years	7	2.7
Position	Chief Executive Officer	23	8.2
	Senior Manager	60	21.6
	Owner of the firm	161	58.5
	Others	32	11.7
Education	Primary School	9	3.2
	High School	74	26.8
	Certificate/Diploma	89	32.3
	Bachelor’s Degree	80	29.1
	Postgraduate Degree	24	8.6
Number of Employees	Less than 5 person	122	43.9
	5–20 person	110	39.9
	21–50 person	34	12.2
	51–100 person	5	2.0
	200 person and more	5	2.0
Income	RM10,000—RM100,000	103	37.4
	RM100,001—RM200,000	93	33.8
	RM200,001—RM300,000	33	11.8
	RM300,001—RM500,000	30	10.7
	RM500,001—RM1,000,000	13	4.5
	RM1,000,001—RM3,000,000	4	1.8
Business Experience	1–2 years	76	27.5
	3–4 years	69	25.0
	5–6 years	51	18.4
	7 years and above	80	29.1
Types of Business	Sole Proprietorship	142	51.5
	Partnership	99	35.9
	Company Act	35	12.6

The results also show that the majority of the entrepreneurs (61.4%) had at least a diploma and/or a degree; the majority had more than 9 years of formal education. As for business experience, 27.5% of respondents had been managing their businesses for 1 to 2 years, 25% for 3 to 4 years, 18.4% for 5 to 6 years, and 29.1% for 7 years or longer.

### Common method variance bias test

Collecting data from a single source might cause issues of common method variance (CMV) bias. Therefore, in order to assess the issues, both procedural and statistical remedies were applied [120]. For procedural remedies, several methods were applied to reduce the likelihood of CMV bias. Specifically, the researcher had improved the scale items and their wording, verified the anonymity and confidentiality of the participants, informed the participants that there were no right or wrong answers, and provided clear instructions on how to complete each section of the questionnaire for respondents [121].

As for a statistical remedy, Harman’s single-factor analysis was applied. Based on this analysis, the existence of the common method is confirmed if the most co-variance explained by the single factor is greater than 40.7% [120]. This research showed no issues of common method bias, because a total of five factors emerged from the Harman’s single-factor analysis and the most co-variance explained by the single factor was only 39.38%, which was less than the threshold value of 40.7%. However, other researchers recommended the construct level correction (CLC) approach for the assessment of the CMV, even though Harman’s single-factor analysis is a commonly used method [122]. The CLC method compares the results of the path coefficient between the original PLS estimation and the CLC estimation to check the existence of common method biases, and it suggests a way to address the CMV [122]. This study applied CMV control constructs (social desirability indicators) as the marker variables after obtaining the path coefficients from the original PLS model constructs to analyze the common method bias. A total of four indicators were adapted from [123] to measure the variable social desirability. The result of the CLC approach indicated that there were no changes between the path coefficients of the original PLS model constructs and the CLC estimations. Therefore, both methods confirmed the absence of CMV in the study.

#### Measurement model analysis

Studies have suggested that researchers analyze the indicator loadings, average variance extracted (AVE), composite reliability, and Cronbach’s alpha values to assess the reliability and validity of the measurement model assessment. Measuring the reliability and validity evaluates whether or not the items represent the same underlying construct. According to Fornell and Larcker (1981) [124], all of the composite reliability values for each latent variable were above 0.7 (see Table 2), confirming the reliability of items for each construct. Moreover, the composite reliability scores ranged from 0.91 to 0.96 and the AVE scores ranged from 0.59 to 0.84, indicating no serious measurement concerns [see 125].

**Table 2:** Measurement model indicating factor loading, composite reliabilities, Cronbach’s alpha, and average variance extracted (n = 276).

First-order construct		Loadings	Cronbach Alpha	AVE	CR
Financial Performance	FinPo2	0.890	0.899	0.832	0.937
	FinPo3	0.928			
	FinPo4	0.918			
Non-financial Performance	NFinPO1	0.773	0.885	0.595	0.911
	NFinPO2	0.795			
	NFINPO3	0.857			
	NFinPO4	0.772			
	NFinPO5	0.736			
	NFinPO6	0.767			
	NFinPO7	0.687			
Bonding	RMOBo1	0.905	0.958	0.799	0.965
	RMOBo2	0.882			
	RMOBo3	0.890			
	RMOBo4	0.893			
	RMOBo5	0.883			
	RMOBo6	0.913			
	RMOBo7	0.889			
Communication	RMOC1	0.914	0.896	0.827	0.935
	RMOC2	0.903			
	RMOC3	0.912			
Empathy	RMOEm1	0.891	0.943	0.815	0.956
	RMOEm2	0.880			
	RMOEm3	0.929			
	RMOEm4	0.902			
	RMOEm5	0.909			
Reciprocity	RMORp1	0.910	0.94	0.847	0.957
	RMORp2	0.921			
	RMORp3	0.925			
	RMORp4	0.925			
Shared value	RMOSh1	0.893	0.934	0.834	0.953
	RMOSh2	0.923			
	RMOSh3	0.923			
	RMOSh4	0.914			
Trust	RMOTR2	0.799	0.897	0.66	0.921
	RMOTr1	0.791			
	RMOTr3	0.846			
	RMOTr4	0.791			
	RMOTr5	0.822			
	RMOTr6	0.824			

Notes: AVE, average variance extracted; CR, composite reliability.

Table 2 shows that the measurement results confirmed the convergent and discriminant validity. As for the standardized factor loadings of all of the constructs, they were above the threshold value of 0.50, and this confirmed the convergent validity of the study [126].

Table 3 shows that the discriminant validity was assessed where the square root of the AVE was compared with the values of the correlations in the respective rows and columns among constructs. The result shows that the square roots of the AVEs were higher than the correlations with any other latent variable, guaranteeing discriminant validity [124].

**Table 3:** Discriminant validity indicating AVE and correlations.

	1	2	3	4	5	6	7	8
1. Bonding	**0.894**							
2.Communication	0.824	**0.910**						
3. Empathy	0.765	0.748	**0.902**					
4. Financial Performance	0.354	0.351	0.381	**0.912**				
5. Nonfinancial Performance	0.538	0.563	0.576	0.619	**0.771**			
6. Reciprocity	0.820	0.806	0.740	0.417	0.601	**0.920**		
7. Shared Values	0.803	0.790	0.752	0.408	0.539	0.822	**0.913**	
8. Trust	0.496	0.473	0.514	0.516	0.718	0.514	0.522	**0.813**

In addition, the loading of each item was greater than all the cross-loadings [127]. Finally, the heterotrait‒monotrait values met the threshold of 0.85 [128]. Overall, the assessments confirmed the reliability and validity of the study, and this indicated that the measurement model of the study was valid and reliably estimated the parameters of the structural model.

#### Structural model analysis

The path analysis was performed to examine the 12 hypotheses of the study. The coefficients of determination (R^2^), path coefficients, and effect sizes (f^2^) of the endogenous latent variables were also calculated [129]. This study applied a 5,000 bootstrap sample from 276 cases to analyze the significance of the findings. The *t*-values (1.65) and *p*-values (0.05) were assessed to test the significance of the hypothesized relationship. The model moderately explained all of the variations in the response variables because the R^2^ value was above the recommended threshold of 0.2 [129]. The direct effects of the main constructs on financial performance and CR (nonfinancial performance) were also tested. Table 4 presents the results of the structural model.

**Table 4:** Hypotheses testing.

	Relationship	Std. Beta	Std.error	t-value	F^2^	Decision
H1a	Trust➔Financial Performance	0.398	0.081	4.932	0.156	Supported
H1b	Bonding➔Financial Performance	-0.109	0.114	0.95	0.004	Not Supported
H1c	Communication➔Financial Performance	-0.039	0.112	0.348	0.001	Not Supported
H1d	Shared value➔Financial Performance	0.113	0.107	1.051	0.004	Not Supported
H1e	Empathy➔Financial Performance	0.058	0.102	0.565	0.002	Not Supported
H1f	Reciprocity➔Financial Performance	0.198	0.111	1.777	0.013	Supported
H2a	Trust➔Customer retention (Non-financial Performance)	0.541	0.048	11.374	0.507	Supported
H2b	Bonding➔Customer retention (Nonfinancial Performance)	-0.089	0.077	1.157	0.004	Not Supported
H2c	Communication➔Customer retention (Nonfinancial Performance)	0.154	0.075	2.06	0.015	Supported
H2d	Shared value➔Customer retention (Non-financial Performance)	-0.118	0.08	1.476	0.009	Not Supported
H2e	Empathy➔Customer retention (Non-financial Performance)	0.147	0.072	2.043	0.018	Supported
H2f	Reciprocity➔Customer retention (Non-financial Performance)	0.26	0.08	3.264	0.039	Supported

**Fig 2:**
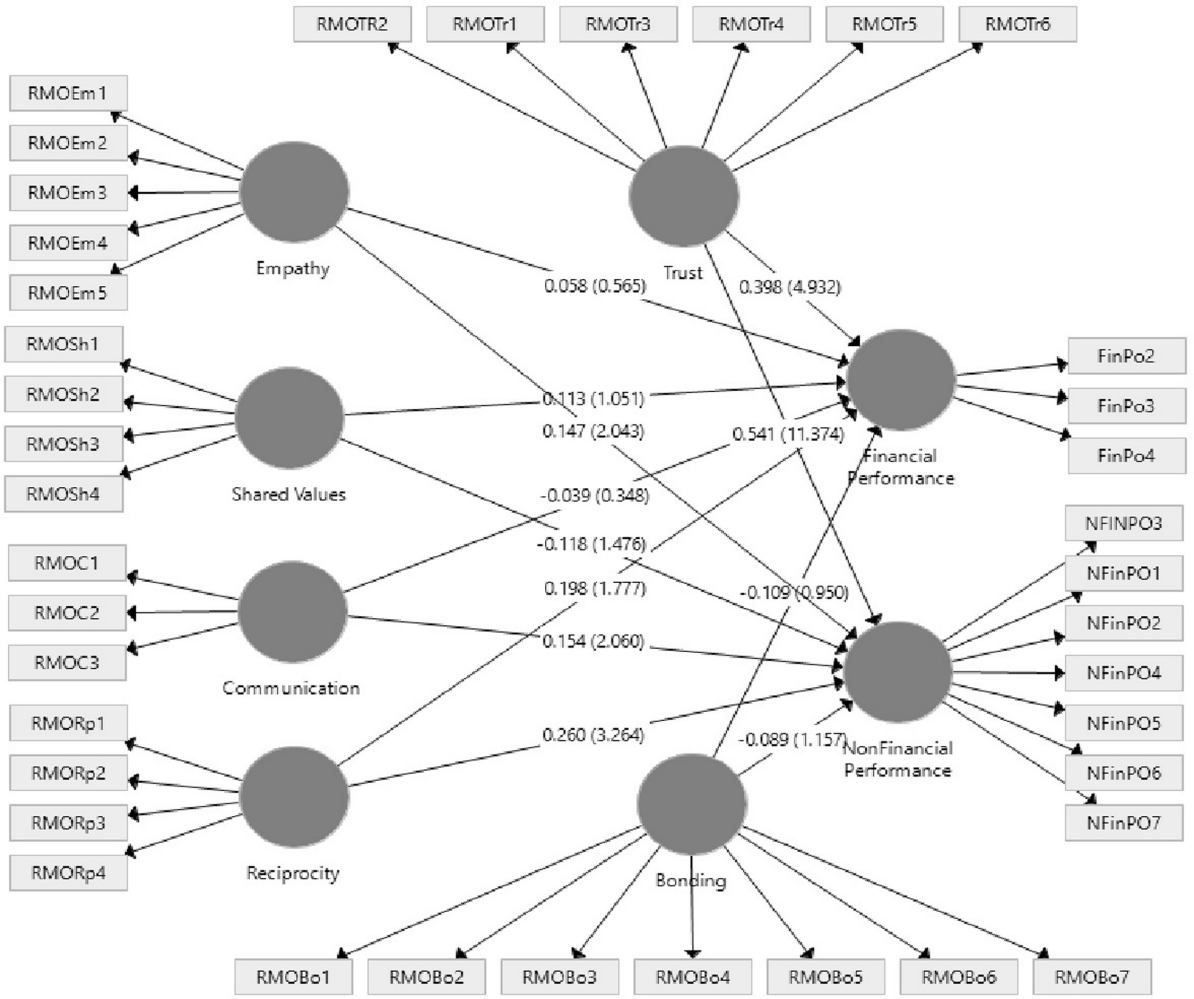
Structural model results.

Fig 2 shows that the research model explains 30.4% of the variance in financial performance. Trust (β = 0.398, *t* = 4.932, *p* < .01) and reciprocity (β = 0.198, *t* = 1.777, *p* < .05) are positively related to financial performance. However, bonding (β = ‒0.109, *t* = 0.950, *p* > .05), communication (β = ‒0.039, *t* = 0.648, *p* > .05), shared values (β = 0.113, *t* = 1.051, *p* > .05), and empathy (β = 0.058, *t* = 0.565, *p* > .05) are not statistically significant. Thus, hypotheses H1a and H1f were supported, whereas hypotheses H1b, H1c, H1d, and H1e were not supported.

The R^2^ value for the endogenous variable of non-financial performance was 60.5%, indicating that the predictor variables could explain 60.5% of the variance in CR. Table 4 shows that trust (β = 0.541, *t* = 11.374, *p* < .01), communication (β = 0.154, *t* = 2.060, *p* < .05), empathy (β = 0.147, *t* = 2.043, *p* < .05), and reciprocity (β = 0.260, *t* = 3.264, *p* < .01) were positively related to CR (non-financial performance). However, bonding (β = ‒0.089, *t* = 1.157, *p* > .05) and shared value (β = ‒0.118, *t* = 1.476, *p* > .05) were not statistically significant. Therefore, hypotheses H2a, H2c, H2e, and H2f were supported, but H2b and H2d were not supported to explain non-financial performance. According to [130], the predictive power of the overall model based on the R^2^ results indicated it was considered as moderate.

## Discussion

The contribution of entrepreneurial support agencies in assisting SMEs has been important in producing more competitive entrepreneurs in the country and helping them to improve the performance of their SMEs. Prior studies revealed the positive influence of RMO on business performance [75,82]. The uniqueness of this study lies in its examination of the relationship between entrepreneur support agency RMO and performance (financial and nonfinancial performances). This study is among the first empirical studies on entrepreneur support agencies and it contributes to a better understanding of the role of entrepreneur support agency RMO in an SME’s financial and nonfinancial performances. This study proposes that the entrepreneur support agency RMO plays a significant role in influencing an SME’s business performance. Based on the RMO theory, social exchange theory, and resource-based view theory, this study hypothesizes that the components of entrepreneur support agency RMO (i.e., trust, bonding, communication, shared values, empathy, and reciprocity) have a positive relationship with an SME’s financial and nonfinancial performances.

A total of 12 hypotheses were tested, and 5 of them were supported. The results of this study reveal that trust and reciprocity have a significant positive impact on an SME’s financial performance and that trust, communication, empathy, and reciprocity have a significant positive impact on an SME’s nonfinancial performance.

The results show that trust was the most significant dimension of financial and nonfinancial performances, followed by reciprocity. The significant relationship between trust and business performance was in line with the findings from prior studies in the literature [e.g., 84,131,132]. Furthermore, trust in the organization has been established as a precursor of firm performance [e.g., 133,134]. Increasing the level of mutual trust between an entrepreneur and a support agency enabled SMEs to establish long-term cooperative relationships and to obtain the necessary business information to enhance the SME’s financial performance. Establishing an environment of trust allowed SMEs to be proactive in meeting market changes and to have a likelihood of success in pursuing their commercial activities.

A significant relationship between reciprocity and financial performance is in accordance with prior studies [75,105,107], indicating that reciprocity exerted an influence on a firm’s market share, sales growth, CR, and return on investment. A strong commitment on the part of entrepreneur support agencies to create investments and help SMEs motivated the SMEs to reciprocate by working harder to improve their business performance.

In spite of the fact that bonding, communication, shared values, and empathy have vital roles in the business sphere, those dimensions were not significantly related to financial performance. The results obtained were somewhat contradictory to those of [27,95,103], who proposed that bonding, shared values, empathy, and communication were associated with performance. Although two-way communication is an effective way for two parties to develop a strong relationship, it does not seem to affect the financial performance of SMEs. A possible explanation for this contradictory finding can be the nature of the situational relationship between support agencies and entrepreneurs. Both are at the organizational level with different working cultures, values, and goals. The main function of the support agencies is to provide support to entrepreneurs indirectly in the form of government-based funding and training without interfering in the daily operation of SMEs. Most entrepreneurs, on the other hand, are private entities for which the main objective is to increase business profitability and business performance. These conflicting goals of support agencies and SMEs create different values. Due to these conflicting values, there was no direct relationship between bonding, shared values, empathy, and communication and the financial performance of SMEs.

The results of this study indicate a significant positive relationship between trust, communication, empathy, and reciprocity and nonfinancial performance. The findings are in line with those of prior studies [e.g., 27,60,75,135], signifying the positive relationship between RMO and components of the nonfinancial performance such as CR. The support agencies that were alert to entrepreneurs’ needs and wants may have increased the SME’s CR. Hence, agencies need to pursue high levels of trust, communication, empathy, and reciprocity in their entrepreneur development programs and activities, so that the SMEs can be sustained and become more competitive.

Contrary to expectations, bonding and shared values were not significantly related to nonfinancial performance. From the relationship marketing perspective, this result contradicts the findings of prior studies [e.g., 27,136,137], indicating that shared values and bonding have a positive and significant influence on retention. A reasonable explanation for these contradictory findings is that the SMEs in this research might have been in an early stage of their relationships (the majority of the respondents had had business experience for only 1 to 2 years). Thus, the effect of bonding and shared values on CR may not have been apparent at this stage.

## Implications

This research contributes to a deeper understanding of the relationship strength concept underpinned by the exchange theory developed by [138–140]. The relationship between an entrepreneur support agency and an entrepreneur is vital in ensuring the success of an SME’s business performance. The developing role of entrepreneur support agencies in expanding SMEs in Malaysia by providing training, consulting services, technological knowledge, and ongoing funding is highly commended. This study offers the first insights into how entrepreneur support agency RMO and an SME’s business performance are related.

The findings of this study emphasize the importance of trust and reciprocity in increasing an SME’s financial and nonfinancial performances. To combat low performance among entrepreneurs who are in an entrepreneur development program, support agencies need to consider the environment of reciprocity and trust as a priority in relationship building. Consequently, support agencies who exhibit this concern are more likely to reduce low sustainability and poor performance among SMEs. These findings are in line with the ideas set out by [89], who proposed that reciprocity from the customer is crucial for future company revenues. This finding provides empirical evidence of prior arguments on stakeholder theory perspective, stating that the firm will gain a competitive advantage and have a better performance if it maintains mutual trust and a cooperative relationship with stakeholders [141,142]. Support agencies should prioritize communication and empathy because of their importance in retaining customers. Communication can be improved by providing information sharing with SMEs either face to face or virtually through a suitable platform.

Regarding its practical implications, the study’s findings provide a significant contribution to the industry because most SMEs are supported by various SME development programs at numerous agencies [143,144]. For example, in Malaysia it is reported that most entrepreneurs have received or been linked to at least one support agency [145,146] and that they received various kinds of assistance, including information on monetary aspects, business premises, training, and market access. In reinforcing the focus of support agency activities, entrepreneurs should engage in interactions with a support agency to make them feel connected with the agency.

## Conclusion

In conclusion, this study advances the knowledge on the roles of entrepreneur support agency RMO (trust, bonding, communication, shared values, empathy and reciprocity) with SMEs’ financial and nonfinancial performance. Empirical data were collected from entrepreneurs who had received assistance from an entrepreneur support agency. This study, in particular, gives insight on the paucity of empirical studies in the existing literature, such as the components of the entrepreneur support agency RMO that may result in positive outcomes such as firm’s performance. The findings confirm that trust and reciprocity have a positive influence on firm’s financial performance. Meanwhile, trust, communication, empathy and reciprocity significantly influence firm’s nonfinancial performance.

It’s worth noting that a good relationship between a support agency and an entrepreneur is more likely to improve SMEs’ long-term viability and performance. The establishment and maintenance of relationships between support agencies and entrepreneurs will provide useful information on the needs of various types of businesses and will allow for the coordination of appropriate assistance and development programs for entrepreneurs, resulting in the most efficient use of resources and public funds. The findings consequently provide a novel view and explanation for the growing awareness among support agencies and entrepreneurs to collaborate on synergistic and win‒win strategic approaches in order to stay competitive, relevant, and sustainable in the market.

## Limitations and future research

A number of important limitations need to be considered. Firstly, the findings of this study are specific to the organizational level of SMEs in Kuala Lumpur and Selangor. Hence, the results of this study may vary if they are tested with different SMEs in different positions and in a different country. Secondly, the findings are based on purposive sampling, and this limits the generalizability of the results. It is suggested that broader global research be undertaken to verify the findings of this study. Thirdly, this study is based on cross-sectional data at one point in time. Most respondents were in the first or second year of business. A longitudinal study should be undertaken to determine the dynamic link between the entrepreneur support agency RMO and the SME’s business performance. Finally, the insignificant findings require further investigation to expand the discussion and provide new insights into the field of relationship marketing between SMEs and entrepreneur support agencies. Hence, a future study in the field of relationship marketing involving support agencies and entrepreneurs could further investigate the links between trust, bonding, communication, shared values, empathy, and reciprocity and the firm performance in a different context to establish and confirm synergies and contradictions.

## Appendix A

**Table:** 

Constructs	Items	Measurement items	References
*Based on the entrepreneur support agency (XYZ) you have received assistance for the past at least one year*, *the entrepreneur supporting agency*, *XYZ………*.			
Trust	RMOTr1	can be trusted.	[27]
	RMOTr2	is reliable in providing unique entrepreneur supporting services/programs.	*Delete RMOTr7*
	RMOTr3	will ensure entrepreneur’s privacy in the service processes.	
	RMOTr4	is consistent in providing quality services to entrepreneurs.	
	RMOTr5	has good reputation.	
	RMOTr6	works in close cooperation with entrepreneurs.	
	RMOTr7	policies and practices are trust worthy.	
Bonding	RMOBo1	tries to establish a long-term relationship with entrepreneurs under XYZ programs.	[27] *Delete RMOBo8 and RMOBo9*
	RMOBo2	works in close operations with entrepreneurs.	
	RMOBo3	keeps in touch with entrepreneurs constantly.	
	RMOBo4	shows a sincere interest in solving problem on time.	
	RMOBo5	tries hard to understand entrepreneurs need constantly.	
	RMOBo6	XYZ employees will search for the best services for entrepreneurs.	
	RMOBo7	XYZ always search for the best solution for entrepreneurs.	
	RMOBo8	and entrepreneurs rely on each other	
	RMOBo9	has well defined standards for entrepreneurs.	
Communication	RMOC1	frequently communicate and express their opinions to each other.	[27]
	RMOC2	entrepreneurs can communicate honestly.	*Delete RMOC4 and RMOC5*
	RMOC3	entrepreneurs can show their discontent towards each other via communication.	
	RMOC4	communicates frequently with the entrepreneurs.	
	RMOC5	executes public relation programs with the entrepreneurs.	
Shared Values			[27] *Accept all 4*
	RMOSh1	share the same worldview with the entrepreneurs.	
	RMOSh2	share the same opinions in many aspects with the entrepreneurs.	
	RMOSh3	share the same values with the entrepreneurs.	
	RMOSh4	share an established relationship with the entrepreneurs because of its good values.	
Empathy	RMOEm1	always look things from entrepreneur’s view.	[27] *Accept all 5*
	RMOEm2	knows how the entrepreneurs feel.	
	RMOEm3	cares about entrepreneur feeling.	
	RMOEm4	gives personal attention to the entrepreneur.	
	RMOEm5	understand the entrepreneur specific needs.	
Reciprocity	RMORp1	“never forget a good turn” as its business slogan.	[27]
	RMORp2	always keeps its promises to others in many situations.	*Delete RMORp5*
	RMORp3	would repay the entrepreneur kindness, if the entrepreneur gave assistance to XYZ’s staff.	
	RMORp4	is generally fair in dealings with the entrepreneur.	
	RMORp5	is willing to do me a favor if asked by the entrepreneur.	
Customer Retention			
*Most of the entrepreneur customers are*: *……*			
	NFinPO1	loyal to the enterprise.	[115–117]
	NFinPO2	want to continue relationship with the enterprise.	*Delete 8 NFinPO8*
	NFinPO3	choose the enterprise as their first choice.	
	NFinPO4	Choose to stay with the enterprise rather than trying a different enterprise that they are unsure of.	
	NFinPO5	intend to continue using the enterprise services over the next few years	
	NFinPO6	committed to the enterprise.	
	NFinPO7	would not change their preference even if their friends were to recommend another enterprise.	
	NFinPO8	care a lot about the enterprise.	
Financial Performance			
*Perceptions on four-financial performance indicators*. *How would you rate your firm’s performance as compared with your competitors on the following from 1 “much worse” to 7 “much better”*?			
	FinPo1 FinPo2 FinPo3 FinPo4	Return on investment Cost Profitability Sales	[118,147] *Delete Fin PO1*

## Supporting information

S1 Data (CSV)
